# Müller-Weiss Syndrome: A Case Report and Review of the Literature

**DOI:** 10.7759/cureus.103486

**Published:** 2026-02-12

**Authors:** Wissal Belhadj, Laila Taoubane, Imane Mohammadine, Abderrahim Majjad, Ahmed Bezza

**Affiliations:** 1 Rheumatology, Hôpital militaire d'instruction Mohammed V, Rabat, MAR; 2 Radiology, Mohammed V Military Training Hospital, Rabat, MAR

**Keywords:** adult-onset osteonecrosis, chronic midfoot pain, foot deformity, müller-weiss syndrome, navicular osteonecrosis

## Abstract

Müller-Weiss syndrome is a rare condition characterized by osteonecrosis of the navicular bone. We describe the case of a 60-year-old woman presenting with chronic, non-traumatic mechanical pain of the right midfoot. Laboratory investigations, including inflammatory markers, were within normal limits. Weight-bearing radiographs and CT demonstrated changes of the navicular bone consistent with Müller-Weiss syndrome. This diagnosis was considered in the context of persistent mechanical midfoot pain, and the patient was managed conservatively, with favorable clinical evolution during follow-up .

## Introduction

Müller-Weiss syndrome is a rare condition characterized by spontaneous osteonecrosis of the navicular bone in adults and represents an uncommon cause of chronic midfoot pain [1]. First described in 1927 by Walther Müller, the disease predominantly affects middle-aged individuals and is frequently underdiagnosed or misattributed to more common degenerative or mechanical foot disorders [2].

Clinically, patients typically present with insidious-onset mechanical midfoot pain, often without a history of trauma, which may lead to delayed diagnosis [3]. Functional impairment during walking and progressive limitation of daily activities are frequently reported, contributing to diagnostic delay in routine clinical practice. As the disease progresses, collapse and deformity of the navicular bone can result in progressive foot deformity and secondary osteoarthritis [4].

Imaging plays a crucial role in diagnosis. Weight-bearing radiographs may demonstrate characteristic changes of the navicular bone, while computed tomography allows better assessment of bone fragmentation and collapse [1,3]. Advanced imaging is also essential to differentiate Müller-Weiss syndrome from more common causes of midfoot pain, such as midfoot osteoarthritis or posterior tibial tendon dysfunction. Early recognition is essential to guide appropriate management and prevent disease progression [5].

Here, we report a case of Müller-Weiss syndrome in a 60-year-old woman presenting with chronic mechanical midfoot pain, followed by a brief review of the literature.

This article was previously presented as a meeting abstract at the 35th National Congress of the Moroccan Society of Rheumatology on May 29-31, 2025.

## Case presentation

A 60-year-old woman presented with an 18-month history of slowly progressive, non-traumatic mechanical pain in the right midfoot. Her medical history was notable for treated ovarian adenocarcinoma and thyroidectomy.

Clinical examination revealed swelling of the midfoot associated with hindfoot varus deformity. Pain was localized to the medial midfoot and exacerbated during weight-bearing. Gait was altered during walking. The hindfoot varus deformity was rigid on clinical examination. There were no clinical signs suggestive of midfoot osteoarthritis or posterior tibial tendon dysfunction. The remainder of the physical examination was unremarkable.

**Figure 1:**
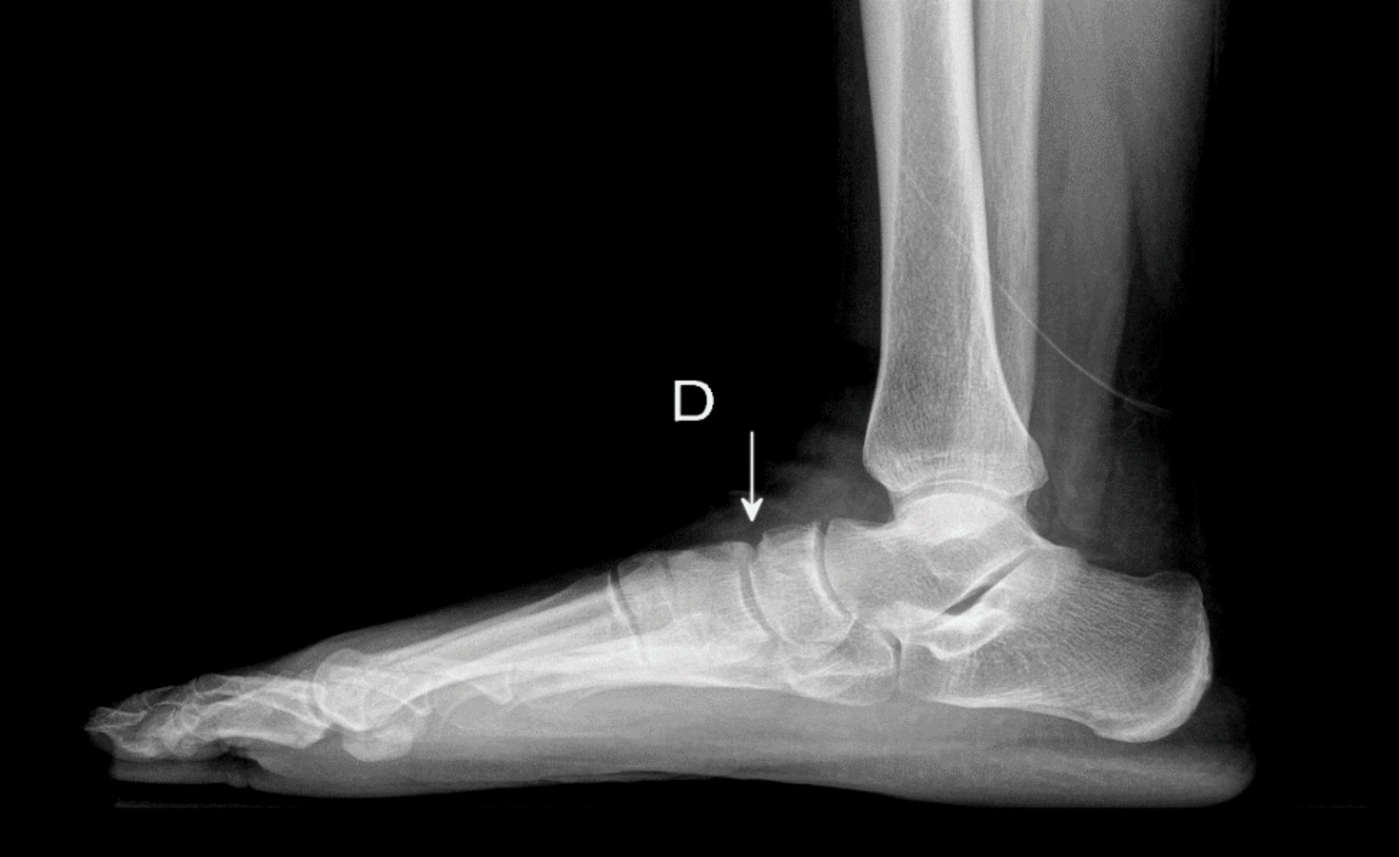
Weight-bearing lateral radiograph of the right foot Localized radiolucent area of the navicular bone (arrow), associated with early bone remodeling and mild flattening, consistent with stage 2 Müller-Weiss syndrome

Standard weight-bearing radiographs and computed tomography of the foot demonstrated localized radiolucency of the navicular bone with early bone remodeling and mild flattening, without significant collapse. These findings were consistent with stage 2 Müller-Weiss syndrome according to the Maceira classification (Figure 1).

Laboratory investigations revealed hypercholesterolemia and hypothyroidism, while inflammatory markers were within normal limits ( Table 1).

**Table 1:** Laboratory findings All laboratory values are expressed using the units of the local laboratory.

Parameter	Result	Reference Range	Unit
C-reactive protein (CRP)	5	<5	mg/L
Erythrocyte sedimentation rate (ESR)	15	<20	mm/h
Thyroid-stimulating hormone (TSH)	5.447	0.4–4.0	mIU/L
Total cholesterol	2.63	<2.0	g/L
Low-density lipoprotein (LDL) cholesterol	1.94	<1.6	g/L
High-density lipoprotein (HDL) cholesterol	0.52	>0.4	g/L
Triglycerides	0.84	<1.5	g/L

Based on clinical and imaging findings, the diagnosis of stage 2 Müller-Weiss syndrome was established. The patient was managed conservatively with joint offloading, analgesic treatment, and orthopedic insoles. Clinical follow-up showed improvement in pain intensity, with a decrease in visual analog scale (VAS) score.

## Discussion

Müller-Weiss syndrome is a rare condition characterized by adult-onset avascular necrosis of the navicular bone and represents an uncommon cause of chronic midfoot pain [1]. Its exact prevalence remains uncertain, likely due to underdiagnosis and delayed recognition, as symptoms are often nonspecific and may be attributed to more common degenerative or mechanical foot disorders [3]. The disease predominantly affects middle-aged women and is frequently bilateral, although unilateral involvement has also been reported [4]. Our patient, a 60-year-old woman presenting with chronic mechanical midfoot pain, fits the typical demographic profile described in the literature.

The etiology of this condition remains unclear and is considered multifactorial. Several hypotheses have been proposed, including altered foot biomechanics, chronic mechanical overload, and vascular insufficiency of the navicular bone, which is particularly vulnerable to central ischemia due to its centripetal blood supply [6,7]. In our patient, the absence of a traumatic history and the insidious onset of symptoms support a non-traumatic, degenerative mechanism consistent with these proposed pathophysiological processes.

Clinically, Müller-Weiss syndrome usually presents with progressive mechanical pain of the midfoot or hindfoot, often leading to delayed diagnosis [5]. Progressive collapse of the navicular bone may result in deformity of the medial longitudinal arch and secondary osteoarthritis [8]. Differentiation from more common conditions such as midfoot osteoarthritis or posterior tibial tendon dysfunction is essential, as clinical presentations may overlap while management strategies differ. Despite an 18-month duration of symptoms in our patient, imaging findings were consistent with an early radiological stage, illustrating the slow and variable progression of the disease, as previously reported [7].

Imaging plays a central role in diagnosis. Weight-bearing radiographs may demonstrate characteristic changes of the navicular bone, while CT allows better assessment of bone remodeling, fragmentation, and collapse, facilitating accurate staging according to the Maceira classification [3,9]. In our case, CT revealed early bone remodeling without fragmentation or talonavicular joint incongruence, and radiographic findings were consistent with stage 2 disease, supporting the choice of conservative management.

Management depends on disease stage and symptom severity. Conservative treatment, including activity modification, offloading, analgesics, and orthotic support, is recommended in early stages and may prevent progression to advanced deformity. Surgical interventions, such as arthrodesis or internal fixation, are generally reserved for advanced stages or cases refractory to conservative treatment [10]. In our patient, conservative management resulted in favorable clinical evolution, with no evidence of radiological progression at follow-up.

## Conclusions

Müller-Weiss syndrome is a rare but clinically important cause of chronic mechanical midfoot pain in adults. Weight-bearing radiography and CT are usually diagnostic. Early recognition allows appropriate conservative management and may prevent progressive foot deformity. In our case, conservative treatment was associated with a favorable clinical response, with improvement in pain intensity at follow-up. Clinicians should consider this entity in patients with unexplained chronic midfoot pain to ensure timely diagnosis and management.
